# Public Health Diplomas

**Published:** 1910-09-03

**Authors:** 


### PUBLIC HEALTH DIPLOMAS.

Diplomas in Public Health are granted by nearly all examining bodies. The requirement for each diploma varies, but particulars may usually be obtained on application to the various registrars. The Conjoint Board of Scotland requires candidates to produce evidence of attendance (subsequent to obtaining a registrable qualification) for six months at a Public Health Laboratory, and for six months under a Medical Officer of Health of a county or of a large urban district. There are two examinations, and candidates may present themselves for both of them at one period or for either examination separately. The fee is £12 12s., or £6 6s. in respect of each examination; and candidates referred are readmitted on a fee of £3 3s. in respect of each examination. Applications for examination in Edinburgh to be sent to Mr. James Robertson, 54 George Square, Edinburgh; and in Glasgow to Mr. Alexander Duncan, B.A., LL.D., 242 St. Vincent Street, Glasgow, not later than fourteen days before the examination day. The University of Dublin confers degrees after examinations upon M.D.s or graduates in Medicine and Surgery of Dublin, Oxford, or Cambridge, and the Royal University of Ireland only on graduates of Medicine of that University. The Conjoint Examining Board grants diplomas after examination to candidates who have complied with the regulations of the General Medical Council and passed the required examinations.

